# Tris[2-(2-pyridylsulfan­yl)eth­yl]ammonium perchlorate

**DOI:** 10.1107/S1600536809052283

**Published:** 2009-12-12

**Authors:** Yan An, Xiao-Feng Li, Hui-Guo Chen, Li-Hua Dong

**Affiliations:** a Institute of Marine Materials Science and Engineering, Shanghai Maritime University, Shanghai 201306, People’s Republic of China

## Abstract

In the title molecular salt, C_21_H_25_N_4_S_3_^+^·ClO_4_^−^, an intra­molecular N—H⋯N hydrogen bond stabilizes the conformation of the cation. The three N—C—C—S torsion angles are 91.7 (2), 100.9 (2) and 167.02 (14)°.

## Related literature

For tripodal ligands as recognition reagents towards small mol­ecules or ions, see: Bretonniere *et al.* (2000 ▶). For a benzene-based tripodal oxazoline as an efficient recognition system for some alkyl­ammonium ions for clinical applications, see: Kim & Ahn (2000 ▶). For the complexation structures and Ln/An selectivities of tripodal *N*-donor ligands, see: Wietzke *et al.* (1998 ▶).
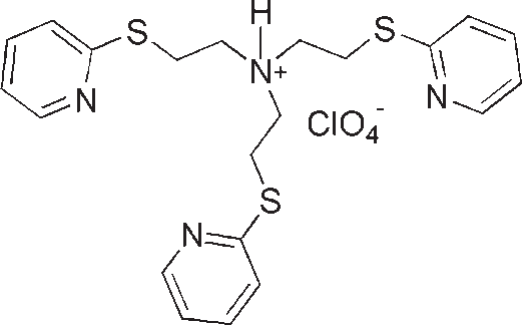


## Experimental

### 

#### Crystal data

C_21_H_25_N_4_S_3_^+^·ClO_4_^−^
*M* *_r_* = 529.08
Triclinic, 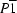

*a* = 8.5480 (7) Å
*b* = 11.9753 (10) Å
*c* = 12.9346 (11) Å
α = 110.884 (4)°
β = 99.609 (4)°
γ = 91.774 (4)°
*V* = 1213.81 (18) Å^3^
*Z* = 2
Mo *K*α radiation
μ = 0.45 mm^−1^
*T* = 296 K
0.37 × 0.35 × 0.33 mm

#### Data collection

Bruker APEXII CCD diffractometer
Absorption correction: multi-scan (*SADABS*; Sheldrick, 2004 ▶) *T*_min_ = 0.846, *T*_max_ = 0.862
6994 measured reflections
4726 independent reflections
3936 reflections with *I* > 2σ(*I*)
*R*_int_ = 0.010

#### Refinement

*R*[*F* ^2^ > 2σ(*F* ^2^)] = 0.040
*wR*(*F* ^2^) = 0.105
*S* = 1.03
4726 reflections
302 parameters
H atoms treated by a mixture of independent and constrained refinement
Δρ_max_ = 0.50 e Å^−3^
Δρ_min_ = −0.43 e Å^−3^

### 

Data collection: *APEX2* (Bruker, 2004 ▶); cell refinement: *SAINT* (Bruker, 2004 ▶); data reduction: *SAINT*; program(s) used to solve structure: *SHELXS97* (Sheldrick, 2008 ▶); program(s) used to refine structure: *SHELXL97* (Sheldrick, 2008 ▶); molecular graphics: *SHELXTL* (Sheldrick, 2008 ▶); software used to prepare material for publication: *SHELXTL*.

## Supplementary Material

Crystal structure: contains datablocks I, global. DOI: 10.1107/S1600536809052283/bx2246sup1.cif

Structure factors: contains datablocks I. DOI: 10.1107/S1600536809052283/bx2246Isup2.hkl

Additional supplementary materials:  crystallographic information; 3D view; checkCIF report

## Figures and Tables

**Table 1:** Hydrogen-bond geometry (Å, °)

*D*—H⋯*A*	*D*—H	H⋯*A*	*D*⋯*A*	*D*—H⋯*A*
N1—H1*N*⋯N2	0.85 (2)	1.98 (2)	2.812 (2)	165 (3)

**Fig. 1.**
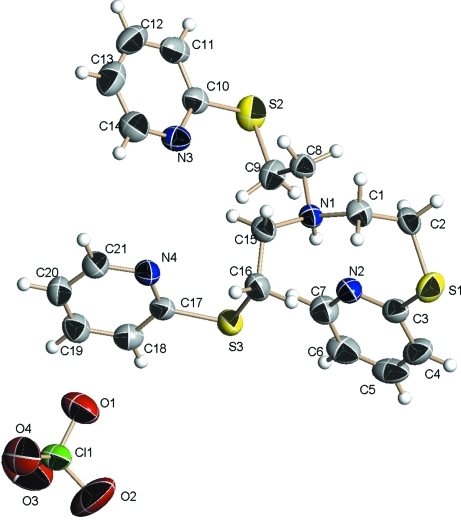
View of the structure of (I). Displacement ellipsoids are drawn at the 30% probability level.

## supplementary crystallographic information

### Comment

A suitably designed tripodal ligand could be an outstanding recognition reagent towards small molecules or ions (Bretonniere *et al.*, 2000). Kim and Ahn developed a benzene-based tripodal oxazoline as an efficient recognition system for some alkylammonium ions for clinical application (Kim & Ahn, 2000). Wietzke *et al.* investigated the complexation structures and Ln/An selectivities of tripodal N-donor ligands (Wietzke *et al.*, 1998). To develop a new type of tripodal ligands which could have good selectivity towards transition metal ions, we reported here the synthesis and the crystal structure of the title compound, which comprises discrete ions which are not interconected, so Coulombic interaction to stabilize the crystal structure. The molecular structure is stabilized by one N— H··· N intramolecular hydrogen bond, Fig 1, Table 1. In the tris(2-(pyridin-2-ylthio)ethyl)ammonium cation , the dihedral angles N-C-C-S are: 91.7 (2) (N1/C1/C2/S1); 100.9 (2) (N1/C15/C16/S3) and 167.02 (14)° (N1/C8/C9/S2). The mean planes of the phenyl rings (A: N4-C17/C21; B: N3-C10/C14; C: N2-C3/C7) make dihedral angles of : 23.85 (12) (between A/C); 89.74 (13) (between B/C) and 71.81 (12)° (between A/B).Two O atoms of perchlorate anion are slightly disordered; it was not split intro two positions.

### Experimental

The title compound was obtained , in an attempts to prepare an coordination compound between tris(2-(pyridin-2-ylthio)ethyl)amine (0.214 g, 0.5 mmol) and Cu(ClO_4_)_2_ (0.132 g, 0.5 mmol) using absolute alcohol (6 ml) as solvent . This mixture was stirred in air for 1 h and the colorless crystals were obtained after evaporating for 5 days. Analysis, calculated for C_21_H_25_N_4_O_4_S_3_Cl: C 47.67, H 4.76, N 10.59%; found: C 47.31, H 4.25, N 10.96%.

### Refinement

H(1N)-atom was located in a difference Fourier and  refined freely. The other H atoms were placed at calculated positions in the riding model approximation (C—H = 0.93 , C—H_2_ = 0.97 Å), with their temperature factors were set to 1.2 times those of the equivalent isotropic temperature factors of the parent atoms.

### Crystal data

**Table:**

C_21_H_25_N_4_S_3_^+^·ClO_4_^−^	*Z* = 2
*M**_r_* = 529.08	*F*(000) = 552
Triclinic, *P*1	*D*_x_ = 1.448 Mg m^−^^3^
Hall symbol: -P 1	Mo *K*α radiation, λ = 0.71073 Å
*a* = 8.5480 (7) Å	Cell parameters from 3657 reflections
*b* = 11.9753 (10) Å	θ = 2.4–28.5°
*c* = 12.9346 (11) Å	µ = 0.45 mm^−^^1^
α = 110.884 (4)°	*T* = 296 K
β = 99.609 (4)°	Block, yellow
γ = 91.774 (4)°	0.37 × 0.35 × 0.33 mm
*V* = 1213.81 (18) Å^3^	

### Data collection

**Table:**

Bruker APEXII CCD diffractometer	4726 independent reflections
Radiation source: fine-focus sealed tube	3936 reflections with *I* > 2σ(*I*)
graphite	*R*_int_ = 0.010
φ and ω scans	θ_max_ = 26.0°, θ_min_ = 1.7°
Absorption correction: multi-scan (*SADABS*; Sheldrick, 2004)	*h* = −10→10
*T*_min_ = 0.846, *T*_max_ = 0.862	*k* = −14→14
6994 measured reflections	*l* = −15→15

### Refinement

**Table:**

Refinement on *F*^2^	Primary atom site location: structure-invariant direct methods
Least-squares matrix: full	Secondary atom site location: difference Fourier map
*R*[*F*^2^ > 2σ(*F*^2^)] = 0.040	Hydrogen site location: inferred from neighbouring sites
*wR*(*F*^2^) = 0.105	H atoms treated by a mixture of independent and constrained refinement
*S* = 1.03	*w* = 1/[σ^2^(*F*_o_^2^) + (0.0436*P*)^2^ + 0.6528*P*] where *P* = (*F*_o_^2^ + 2*F*_c_^2^)/3
4726 reflections	(Δ/σ)_max_ = 0.001
302 parameters	Δρ_max_ = 0.50 e Å^−^^3^
0 restraints	Δρ_min_ = −0.42 e Å^−^^3^

### Special details

**Table:**

Geometry. All e.s.d.'s (except the e.s.d. in the dihedral angle between two l.s. planes) are estimated using the full covariance matrix. The cell e.s.d.'s are taken into account individually in the estimation of e.s.d.'s in distances, angles and torsion angles; correlations between e.s.d.'s in cell parameters are only used when they are defined by crystal symmetry. An approximate (isotropic) treatment of cell e.s.d.'s is used for estimating e.s.d.'s involving l.s. planes.
Refinement. Refinement of *F*^2^ against ALL reflections. The weighted *R*-factor *wR* and goodness of fit *S* are based on *F*^2^, conventional *R*-factors *R* are based on *F*, with *F* set to zero for negative *F*^2^. The threshold expression of *F*^2^ > σ(*F*^2^) is used only for calculating *R*-factors(gt) *etc*. and is not relevant to the choice of reflections for refinement. *R*-factors based on *F*^2^ are statistically about twice as large as those based on *F*, and *R*- factors based on ALL data will be even larger.

### Fractional atomic coordinates and isotropic or equivalent isotropic displacement parameters (Å^2^)

**Table:**

	*x*	*y*	*z*	*U*_iso_*/*U*_eq_	
C1	0.6881 (3)	0.46034 (19)	0.84074 (18)	0.0474 (5)	
H1A	0.5787	0.4756	0.8204	0.057*	
H1B	0.7181	0.4941	0.9223	0.057*	
C2	0.6983 (3)	0.32631 (19)	0.80047 (18)	0.0496 (5)	
H2A	0.6785	0.2988	0.8595	0.060*	
H2B	0.8064	0.3111	0.7898	0.060*	
C3	0.6641 (3)	0.24033 (19)	0.56478 (18)	0.0474 (5)	
C4	0.6269 (3)	0.1444 (2)	0.4618 (2)	0.0632 (6)	
H4	0.5527	0.0810	0.4512	0.076*	
C5	0.7022 (4)	0.1453 (2)	0.3761 (2)	0.0703 (8)	
H5	0.6791	0.0825	0.3064	0.084*	
C6	0.8115 (3)	0.2394 (2)	0.3942 (2)	0.0656 (7)	
H6	0.8643	0.2415	0.3375	0.079*	
C7	0.8414 (3)	0.3303 (2)	0.49763 (19)	0.0569 (6)	
H7	0.9161	0.3939	0.5097	0.068*	
C8	0.9644 (2)	0.53834 (19)	0.85158 (17)	0.0443 (5)	
H8A	0.9845	0.4716	0.8762	0.053*	
H8B	0.9814	0.6115	0.9181	0.053*	
C9	1.0806 (3)	0.5451 (2)	0.77764 (19)	0.0532 (5)	
H9A	1.0468	0.5992	0.7394	0.064*	
H9B	1.0820	0.4662	0.7208	0.064*	
C10	1.2649 (3)	0.7542 (2)	0.91802 (19)	0.0487 (5)	
C11	1.3855 (3)	0.8231 (3)	1.0063 (2)	0.0660 (7)	
H11	1.4706	0.7883	1.0335	0.079*	
C12	1.3746 (4)	0.9446 (3)	1.0520 (2)	0.0781 (9)	
H12	1.4517	0.9935	1.1124	0.094*	
C13	1.2506 (4)	0.9930 (3)	1.0086 (2)	0.0755 (8)	
H13	1.2421	1.0752	1.0380	0.091*	
C14	1.1389 (3)	0.9186 (2)	0.9210 (2)	0.0643 (6)	
H14	1.0550	0.9525	0.8914	0.077*	
C15	0.7377 (3)	0.64094 (18)	0.79479 (18)	0.0481 (5)	
H15A	0.8273	0.6933	0.7957	0.058*	
H15B	0.6980	0.6784	0.8639	0.058*	
C16	0.6077 (3)	0.6288 (2)	0.6955 (2)	0.0555 (6)	
H16A	0.5502	0.6998	0.7150	0.067*	
H16B	0.5330	0.5602	0.6819	0.067*	
C17	0.7470 (2)	0.75988 (19)	0.58998 (17)	0.0447 (5)	
C18	0.7867 (3)	0.7806 (2)	0.49839 (19)	0.0523 (5)	
H18	0.7791	0.7180	0.4293	0.063*	
C19	0.8374 (3)	0.8954 (2)	0.5120 (2)	0.0577 (6)	
H19	0.8631	0.9124	0.4518	0.069*	
C20	0.8496 (3)	0.9850 (2)	0.6158 (2)	0.0580 (6)	
H20	0.8848	1.0636	0.6276	0.070*	
C21	0.8088 (3)	0.9557 (2)	0.7016 (2)	0.0565 (6)	
H21	0.8176	1.0168	0.7717	0.068*	
Cl1	0.81316 (7)	0.76318 (5)	0.16119 (4)	0.05665 (17)	
N1	0.7943 (2)	0.52226 (15)	0.79143 (14)	0.0386 (4)	
N2	0.7689 (2)	0.33273 (15)	0.58241 (14)	0.0462 (4)	
N3	1.1428 (2)	0.79961 (17)	0.87518 (15)	0.0518 (4)	
N4	0.7570 (2)	0.84479 (16)	0.69109 (15)	0.0509 (4)	
O1	0.9039 (3)	0.73699 (19)	0.25035 (17)	0.0908 (7)	
O2	0.6898 (4)	0.6723 (2)	0.0992 (3)	0.1414 (12)	
O3	0.9173 (4)	0.7694 (3)	0.0890 (2)	0.1406 (12)	
O4	0.7508 (3)	0.87338 (17)	0.20429 (19)	0.0944 (7)	
S1	0.56374 (8)	0.23770 (6)	0.67216 (6)	0.06351 (19)	
S2	1.27812 (8)	0.59780 (6)	0.86105 (7)	0.0685 (2)	
S3	0.67777 (9)	0.61051 (5)	0.56746 (5)	0.06103 (18)	
H1N	0.791 (3)	0.475 (2)	0.724 (2)	0.050 (6)*	

### Atomic displacement parameters (Å^2^)

**Table:**

	*U*^11^	*U*^22^	*U*^33^	*U*^12^	*U*^13^	*U*^23^
C1	0.0515 (12)	0.0535 (12)	0.0436 (11)	0.0084 (10)	0.0196 (9)	0.0205 (9)
C2	0.0546 (13)	0.0534 (12)	0.0505 (12)	0.0039 (10)	0.0125 (10)	0.0295 (10)
C3	0.0491 (12)	0.0422 (11)	0.0473 (11)	0.0100 (9)	0.0029 (9)	0.0143 (9)
C4	0.0681 (16)	0.0439 (12)	0.0605 (15)	0.0037 (11)	−0.0066 (12)	0.0071 (11)
C5	0.0872 (19)	0.0593 (15)	0.0434 (13)	0.0265 (14)	−0.0040 (13)	−0.0003 (11)
C6	0.0836 (18)	0.0672 (16)	0.0425 (12)	0.0241 (14)	0.0161 (12)	0.0128 (11)
C7	0.0699 (15)	0.0554 (13)	0.0446 (12)	0.0092 (11)	0.0172 (11)	0.0144 (10)
C8	0.0481 (12)	0.0448 (11)	0.0398 (10)	0.0066 (9)	0.0090 (9)	0.0149 (9)
C9	0.0572 (13)	0.0457 (12)	0.0538 (13)	0.0053 (10)	0.0207 (11)	0.0103 (10)
C10	0.0415 (11)	0.0615 (13)	0.0503 (12)	0.0014 (10)	0.0126 (9)	0.0276 (10)
C11	0.0460 (13)	0.098 (2)	0.0656 (15)	−0.0117 (13)	0.0021 (11)	0.0491 (15)
C12	0.082 (2)	0.088 (2)	0.0523 (15)	−0.0362 (17)	0.0042 (14)	0.0187 (14)
C13	0.093 (2)	0.0581 (16)	0.0667 (17)	−0.0100 (15)	0.0238 (16)	0.0102 (13)
C14	0.0744 (17)	0.0574 (15)	0.0598 (15)	0.0143 (13)	0.0158 (13)	0.0181 (12)
C15	0.0603 (13)	0.0386 (11)	0.0450 (11)	0.0110 (9)	0.0097 (10)	0.0144 (9)
C16	0.0539 (13)	0.0546 (13)	0.0635 (14)	0.0028 (10)	0.0041 (11)	0.0316 (11)
C17	0.0435 (11)	0.0458 (11)	0.0434 (11)	0.0053 (9)	0.0009 (9)	0.0179 (9)
C18	0.0502 (12)	0.0589 (13)	0.0450 (12)	0.0086 (10)	0.0101 (10)	0.0151 (10)
C19	0.0571 (14)	0.0680 (15)	0.0571 (14)	0.0037 (11)	0.0174 (11)	0.0312 (12)
C20	0.0588 (14)	0.0506 (13)	0.0671 (15)	−0.0020 (11)	0.0086 (12)	0.0265 (12)
C21	0.0683 (15)	0.0466 (12)	0.0492 (12)	0.0028 (11)	0.0064 (11)	0.0137 (10)
Cl1	0.0721 (4)	0.0453 (3)	0.0425 (3)	0.0206 (3)	0.0058 (3)	0.0051 (2)
N1	0.0486 (10)	0.0367 (8)	0.0307 (8)	0.0061 (7)	0.0114 (7)	0.0107 (7)
N2	0.0536 (10)	0.0424 (9)	0.0399 (9)	0.0065 (8)	0.0100 (8)	0.0112 (7)
N3	0.0539 (11)	0.0553 (11)	0.0431 (10)	0.0090 (9)	0.0077 (8)	0.0149 (8)
N4	0.0639 (12)	0.0463 (10)	0.0417 (9)	0.0041 (8)	0.0065 (8)	0.0166 (8)
O1	0.1193 (18)	0.0864 (14)	0.0666 (12)	0.0355 (13)	0.0050 (12)	0.0317 (11)
O2	0.126 (2)	0.0691 (15)	0.160 (3)	−0.0060 (14)	−0.041 (2)	−0.0091 (16)
O3	0.174 (3)	0.184 (3)	0.0901 (18)	0.067 (2)	0.075 (2)	0.0556 (19)
O4	0.1193 (18)	0.0551 (11)	0.0962 (15)	0.0403 (11)	0.0168 (13)	0.0118 (10)
S1	0.0563 (4)	0.0638 (4)	0.0661 (4)	−0.0120 (3)	0.0106 (3)	0.0206 (3)
S2	0.0469 (3)	0.0638 (4)	0.1021 (5)	0.0159 (3)	0.0218 (3)	0.0347 (4)
S3	0.0852 (5)	0.0449 (3)	0.0473 (3)	−0.0027 (3)	−0.0015 (3)	0.0172 (2)

### Geometric parameters (Å, °)

**Table:**

C1—N1	1.506 (3)	C12—C13	1.358 (4)
C1—C2	1.511 (3)	C12—H12	0.9300
C1—H1A	0.9700	C13—C14	1.362 (4)
C1—H1B	0.9700	C13—H13	0.9300
C2—S1	1.798 (2)	C14—N3	1.337 (3)
C2—H2A	0.9700	C14—H14	0.9300
C2—H2B	0.9700	C15—N1	1.504 (2)
C3—N2	1.330 (3)	C15—C16	1.512 (3)
C3—C4	1.393 (3)	C15—H15A	0.9700
C3—S1	1.758 (2)	C15—H15B	0.9700
C4—C5	1.375 (4)	C16—S3	1.797 (2)
C4—H4	0.9300	C16—H16A	0.9700
C5—C6	1.368 (4)	C16—H16B	0.9700
C5—H5	0.9300	C17—N4	1.327 (3)
C6—C7	1.368 (3)	C17—C18	1.385 (3)
C6—H6	0.9300	C17—S3	1.772 (2)
C7—N2	1.339 (3)	C18—C19	1.369 (3)
C7—H7	0.9300	C18—H18	0.9300
C8—N1	1.502 (3)	C19—C20	1.372 (3)
C8—C9	1.508 (3)	C19—H19	0.9300
C8—H8A	0.9700	C20—C21	1.369 (3)
C8—H8B	0.9700	C20—H20	0.9300
C9—S2	1.804 (2)	C21—N4	1.339 (3)
C9—H9A	0.9700	C21—H21	0.9300
C9—H9B	0.9700	Cl1—O4	1.3980 (18)
C10—N3	1.323 (3)	Cl1—O2	1.402 (2)
C10—C11	1.389 (3)	Cl1—O3	1.411 (3)
C10—S2	1.766 (2)	Cl1—O1	1.416 (2)
C11—C12	1.375 (4)	N1—H1N	0.85 (2)
C11—H11	0.9300		
			
N1—C1—C2	112.63 (16)	C14—C13—H13	120.7
N1—C1—H1A	109.1	N3—C14—C13	123.9 (3)
C2—C1—H1A	109.1	N3—C14—H14	118.0
N1—C1—H1B	109.1	C13—C14—H14	118.0
C2—C1—H1B	109.1	N1—C15—C16	112.76 (17)
H1A—C1—H1B	107.8	N1—C15—H15A	109.0
C1—C2—S1	116.00 (16)	C16—C15—H15A	109.0
C1—C2—H2A	108.3	N1—C15—H15B	109.0
S1—C2—H2A	108.3	C16—C15—H15B	109.0
C1—C2—H2B	108.3	H15A—C15—H15B	107.8
S1—C2—H2B	108.3	C15—C16—S3	114.41 (17)
H2A—C2—H2B	107.4	C15—C16—H16A	108.7
N2—C3—C4	122.2 (2)	S3—C16—H16A	108.7
N2—C3—S1	120.20 (16)	C15—C16—H16B	108.7
C4—C3—S1	117.62 (19)	S3—C16—H16B	108.7
C5—C4—C3	118.6 (2)	H16A—C16—H16B	107.6
C5—C4—H4	120.7	N4—C17—C18	123.8 (2)
C3—C4—H4	120.7	N4—C17—S3	119.39 (16)
C6—C5—C4	119.4 (2)	C18—C17—S3	116.78 (16)
C6—C5—H5	120.3	C19—C18—C17	118.4 (2)
C4—C5—H5	120.3	C19—C18—H18	120.8
C7—C6—C5	118.4 (2)	C17—C18—H18	120.8
C7—C6—H6	120.8	C18—C19—C20	119.1 (2)
C5—C6—H6	120.8	C18—C19—H19	120.5
N2—C7—C6	123.6 (2)	C20—C19—H19	120.5
N2—C7—H7	118.2	C21—C20—C19	118.3 (2)
C6—C7—H7	118.2	C21—C20—H20	120.8
N1—C8—C9	112.11 (17)	C19—C20—H20	120.8
N1—C8—H8A	109.2	N4—C21—C20	124.3 (2)
C9—C8—H8A	109.2	N4—C21—H21	117.8
N1—C8—H8B	109.2	C20—C21—H21	117.8
C9—C8—H8B	109.2	O4—Cl1—O2	110.19 (16)
H8A—C8—H8B	107.9	O4—Cl1—O3	109.95 (18)
C8—C9—S2	110.37 (16)	O2—Cl1—O3	108.4 (2)
C8—C9—H9A	109.6	O4—Cl1—O1	109.82 (13)
S2—C9—H9A	109.6	O2—Cl1—O1	111.28 (18)
C8—C9—H9B	109.6	O3—Cl1—O1	107.10 (17)
S2—C9—H9B	109.6	C8—N1—C15	110.80 (16)
H9A—C9—H9B	108.1	C8—N1—C1	110.78 (15)
N3—C10—C11	123.5 (2)	C15—N1—C1	111.01 (16)
N3—C10—S2	119.08 (17)	C8—N1—H1N	107.1 (16)
C11—C10—S2	117.41 (19)	C15—N1—H1N	110.9 (16)
C12—C11—C10	117.6 (3)	C1—N1—H1N	106.1 (15)
C12—C11—H11	121.2	C3—N2—C7	117.69 (19)
C10—C11—H11	121.2	C10—N3—C14	116.7 (2)
C13—C12—C11	119.7 (3)	C17—N4—C21	116.07 (19)
C13—C12—H12	120.2	C3—S1—C2	104.27 (10)
C11—C12—H12	120.2	C10—S2—C9	101.18 (11)
C12—C13—C14	118.6 (3)	C17—S3—C16	102.03 (11)
C12—C13—H13	120.7		
			
N1—C1—C2—S1	−88.3 (2)	C16—C15—N1—C1	−84.1 (2)
N2—C3—C4—C5	0.4 (3)	C2—C1—N1—C8	−78.5 (2)
S1—C3—C4—C5	179.12 (19)	C2—C1—N1—C15	157.92 (18)
C3—C4—C5—C6	0.3 (4)	C4—C3—N2—C7	−1.1 (3)
C4—C5—C6—C7	−0.4 (4)	S1—C3—N2—C7	−179.76 (17)
C5—C6—C7—N2	−0.3 (4)	C6—C7—N2—C3	1.1 (3)
N1—C8—C9—S2	167.02 (14)	C11—C10—N3—C14	0.2 (3)
N3—C10—C11—C12	1.1 (3)	S2—C10—N3—C14	−179.71 (17)
S2—C10—C11—C12	−179.07 (18)	C13—C14—N3—C10	−1.0 (4)
C10—C11—C12—C13	−1.5 (4)	C18—C17—N4—C21	0.1 (3)
C11—C12—C13—C14	0.7 (4)	S3—C17—N4—C21	−179.39 (17)
C12—C13—C14—N3	0.6 (4)	C20—C21—N4—C17	0.4 (4)
N1—C15—C16—S3	−79.1 (2)	N2—C3—S1—C2	−27.5 (2)
N4—C17—C18—C19	−0.8 (3)	C4—C3—S1—C2	153.81 (18)
S3—C17—C18—C19	178.63 (18)	C1—C2—S1—C3	85.25 (17)
C17—C18—C19—C20	1.1 (3)	N3—C10—S2—C9	−13.36 (19)
C18—C19—C20—C21	−0.6 (4)	C11—C10—S2—C9	166.76 (17)
C19—C20—C21—N4	−0.2 (4)	C8—C9—S2—C10	−79.99 (17)
C9—C8—N1—C15	−82.6 (2)	N4—C17—S3—C16	9.5 (2)
C9—C8—N1—C1	153.72 (17)	C18—C17—S3—C16	−170.05 (17)
C16—C15—N1—C8	152.34 (18)	C15—C16—S3—C17	−79.31 (18)

### Hydrogen-bond geometry (Å, °)

**Table:**

*D*—H···*A*	*D*—H	H···*A*	*D*···*A*	*D*—H···*A*
N1—H(1N)···N2	0.85 (2)	1.98 (2)	2.812 (2)	165 (3)
